# The impact of nitrogen gas flushing on the stability of seasonings: volatile compounds and sensory perception of cheese & onion seasoned potato crisps†
†Electronic supplementary information (ESI) available. See DOI: 10.1039/c8fo00817e


**DOI:** 10.1039/c8fo00817e

**Published:** 2018-08-10

**Authors:** Deepa Agarwal, Lim Mui, Emma Aldridge, Richard Mottram, James McKinney, Ian D. Fisk

**Affiliations:** a School of Bioscience , University of Nottingham , Sutton Bonington , Loughborough , LE12 5RD , UK . Email: ian.fisk@nottingham.ac.uk; b Pipers Crisps Ltd , Pegasus Road , Elsham Wold , Brigg , Lincolnshire DN20 0SQ , UK

## Abstract

The impact of natural cheese and onion seasoning on the sensory perception and shelf life stability of potato crisps was studied. This study was carried out to evaluate the impact of nitrogen (N_2_) gas flushing on the shelf-life stability and sensory attributes of cheese & onion seasoned potato crisps. Markers of lipid oxidation and flavour volatile compounds were evaluated in sliced, unwashed, batch fried potato crisps during accelerated storage at 45 °C for 10 weeks. Volatile flavour compounds were evaluated by headspace gas analysis using solid phase microextraction (SPME) GC-MS and correlated with sensory perception. Gas flushing significantly increases the shelf life of both unsalted and cheese & onion seasoned potato crisps in terms of the stability of volatile compounds and sensory perception. Further to this, the addition of the cheese & onion seasoning increased the shelf life of the base potato crisp; this is proposed to be explained by the antioxidant properties of milk proteins in the seasoning. Sensory evaluation indicated that the key negative sensory attributes reported were rancidity and stale attributes, which were correlated to the progression of lipid oxidation and loss of flavour over storage time irrespective of the packaging environment (with/without gas flushing).

## Abstract 2

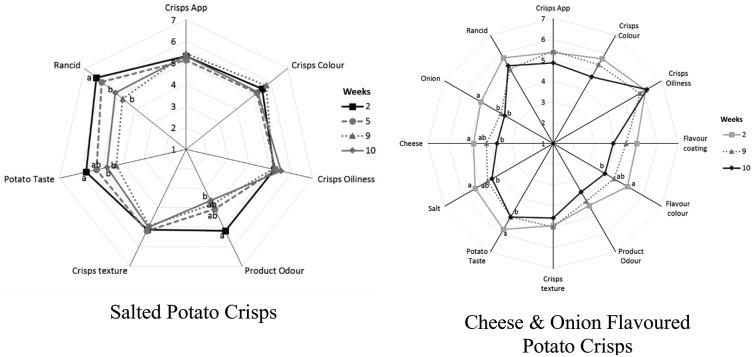
Flavoured potato crisps is the UK's most consumed snack food product. Addition of N_2_ gas flushing extended shelf life by 2 weeks and the addition of seasoning further enhanced the shelf life of the base crisps by 2 weeks under accelerated conditions.

## 1. Introduction

Typically, potato crisps are produced by deep-frying fresh potato slices in a vegetable/sunflower oil bath followed by adding flavour/seasoning to the fried potato crisps. The shelf life of the seasoned potato crisps is dependent on multiple interacting components present in the system. Different physical and chemical factors are known to directly affect oxidative stability (development of rancidity); these include oxygen content, temperature, metal catalysis, light and seasoning stability itself added to potato crisps in terms of oxidation and taste perception.^1^–^4^

Autoxidation is a spontaneous reaction of molecular oxygen with lipids (edible oils), leading to oxidative deterioration.^4^ Edible oil is oxidised by triplet oxygen (^3^O_2_) forming lipid hydroperoxides.^5^ For example during the autoxidation of oleate (in high oleic oils), four allylic hydroperoxides containing –OOH groups on carbon 8 and 11 (*cis*-8-OOH, *cis*-11-OOH) and 9 and 10, (*trans*-9-OOH, *trans*-10-OOH) are produced.^6^–^8^ Hydroperoxides, the primary oxidation products, are unstable and easily decompose^9^ to form products such as aldehydes, ketones, alcohols, hydrocarbons, volatile organic acids, and epoxy compounds and these compounds are known as secondary oxidation products,^5^,^10^ which contribute to the recognisable unpleasant odour of oxidised oil.^10^–^12^ Silva (2005) identified a total of 31 compounds (ketones, aldehydes and alcohols) in oxidised potato crisps using GCMS-SPME extraction procedure.^13^ These oxidised compounds result mainly from the degradation/rearrangement of lipids and carbohydrates present in potato crisps. Hexanal is used as a common marker of oxidation progression as a major product of the oxidation of fats and is known to increase over storage.^14^ Autoxidation of oils in crisps products is a relatively slow process at ambient temperature; however, it is accelerated by elevated temperature.^3^,^15^,^16^ ASLT involves subjecting the product to two or three given constant environmental conditions and measuring the rate of quality loss for each condition. An important concept in ASLT is *Q*_10_, *i.e.* it is the increase in the rate of a reaction when the temperature is increased by 10 °C.1
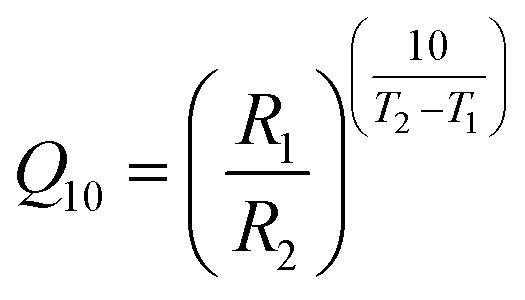



Accelerated shelf life testing can be expressed using the *Q*_10_ concept. *Q*_10_ is the increase in the rate of a reaction (*R*) when the temperature (*T*) is increased by 10 °C.^6^,^17^ For example, 1-week storage at 45 °C is equivalent to 2 weeks of shelf life at 35 °C and 4 weeks at ambient temperature.^17^

Flavour stability in a seasoned snack food such as potato crisps is of the great importance because of its relationship with the quality and acceptability of foods over the shelf life but is often difficult to control. With growing consumer concerns about the source and authenticity of different ingredients in the seasoning, there is increasing demand for the food industry to formulate seasonings with all natural ingredients. Depending on the seasoning formulation the shelf life stability may vary. Besides formulations, there are various factors that impact stability, such as different manufacturing and storage processes and packaging materials. These cause modifications in the overall flavour profile either by reducing aroma compound intensity in seasonings or by producing off-flavour compounds.^18^,^19^ For well-seasoned potato crisps, both the lipid oxidation and flavour-acceptability should be evaluated and controlled over the shelf life of the product. Besides the importance of the shelf life stability of the natural seasoning for the snack food products, little is known, or to the best of authors’ knowledge, there are no studies investigating the effect of natural seasoning on the shelf life stability of the base (unseasoned) product.

Autoxidation of oil takes place at an accelerated rate in the presence of oxygen and metal ions,^20^ where both the concentration and type of oxygen affects the oxidation of oils.^16^ Andersson (1998) reported that the oxygen concentration in the oil is dependent on the oxygen partial pressure in the headspace of the oil, where a higher amount of oxygen is dissolved in the oil when the headspace oxygen partial pressure increases, leading to a higher rate of oxidation.^20^,^21^ In recent years, modified atmosphere packaging (MAP) methods are increasingly being used to lower the oxygen concentration and thereby reduce the rate of oxidative degradation.^22^ Modified atmosphere packaging (MAP) is defined by Ahvenainen (1995) as a method where the normal gas atmosphere is changed in a package headspace by a single gas or a combination of gases to balance safe shelf life extension.^23^ Typically for a dry oily snack food such as potato crisps, nitrogen gas (N_2_) can be therefore used to increase the shelf life.

Analysis of lipid oxidation in food products is a difficult process due to its complex nature and number of components in food products interfering with the analysis; for example, commonly natural antioxidants are added in the food products, which tends to complicate the process to monitor the progression of lipid oxidation.^1^,^2^,^4^,^5^ There are several standard chemical and physical methods to assess the quality of fats and fat-containing food products, peroxide value (PV), TBA (thiobarbituric acid), oxygen absorption, headspace analysis of volatiles and most commonly sensory analysis.^2^,^5^,^8^,^13^ In recent years, isolation of volatiles associated with lipid oxidation in static as well as dynamic headspace methods is widely used, especially in cases where high sensitivity is needed to monitor the different compounds associated with product instability.^24^ Headspace chromatographic techniques such as SPME (solid phase microextraction) have gained popularity in the food industry for lipid oxidation of volatiles and volatiles associated with other components in the product for quality controls due to the simplicity, robustness, low cost, and high sensitivity compared to other chemical methods.^25^

The primary aim of this study then is focused on understanding the impact of seasoning produced from all-natural ingredients on the sensory perception and shelf life stability of well-seasoned potato crisps. A detailed screening of different volatile compounds in well-seasoned potato crisps *i.e.* cheese & onion seasoned potato crisps and unsalted potato crisps was performed. The second objective of the work was to monitor the development of volatile compounds associated with seasoning along with lipid oxidation and impact of nitrogen gas flushing over accelerated shelf-life storage. A detailed study of volatiles profiles associated with both seasoning and lipid oxidation in correlation with sensory perception of the product, as presented here, will enable important insight into the complexity of the well-seasoned potato crisps/other food products produced from all natural resources. This study is unique as it is a first attempt to investigate the impact of nitrogen flushing and seasoning addition on the shelf life of well-seasoned potato crisps where both potato/oil oxidation and flavour stability were tested together and correlated with sensory perception.

## 2. Materials and methods

### 2.1. Materials

Unsalted potato crisps and cheese & onion flavour potato crisps were supplied by Pipers Crisps Ltd (Lincoln, UK), where 4 batches (2 batches with N_2_ gas flushing and 2 batches without gas flushing, from each batch of 6 boxes sized 40 × 40 g) of each product were supplied. Cheese & onion flavour produced using all natural ingredients was supplied by Frutarom (UK). The basic composition of unsalted potato crisps for the 100 g pack is energy 553 Kcal, carbohydrates 56.1% of which sugars are 0.6%, protein 4.9%, fibre 3.8%, fat 33% (of which saturated 3.5%) and salt 0.06%. 3-Heptanone used as an internal standard during SPME-GC-MS analysis was supplied by Sigma-Aldrich (UK).

### 2.2. Flavour potato crisps preparation

Freshly fried potato crisps were obtained from commercial batch frying units (Pipers Crisps Ltd, UK), and were fried in 100% high oleic sunflower oil (HOSO) (Kerfoot, Goole, UK) before being mixed with a cheese & onion seasoning. 8% w/w cheese & onion seasoning (Pipers Crisps standard flavour application rate to maintain the total salt level (1.55 g per 100 g) in the final product) was added to potato crisps and mixed well using an industrial flavour coating rotating drum. A batch each of unsalted and cheese & onion was flushed with nitrogen gas before sealing the packets, with a residual oxygen content of below 3.5%; all samples were produced on the same production date and Pipers Crisps had full traceability of the product. Throughout the crisps frying process free fatty acid (FFA) content below 0.5% and total polar material (TPM) below 15% were maintained, to control the chemical quality of the frying oil. Since there is no legal limit in the UK, EU regulations suggest that for deep-fried products when oil reaches 24% TPM, the frying life should be terminated and FFA should be maintained as low as possible typically below 0.5% due to the possible impact on the taste.^25^,^29^ All the cheese and onion seasoned and unsalted potato crisps samples were packed in plain aluminium foil with 20 Matt OPP/40 Met OPP Laminate film and sealed.

### 2.3. Accelerated shelf life sample preparation

All sealed bags of unsalted and cheese & onion seasoned potato crisps both gas-flushed or non-gas flushed were stored in closed chambers at 45 °C until further analysis. Additional samples were stored at 25 °C and 35 °C for more accurate shelf life predictions (data not shown). Bags were collected at fixed intervals (2, 3, 5, 7, 9 and 10 weeks) and stored individually in airtight containers at –80 °C prior to analysis. Reference samples (freshly prepared samples at ambient temperature) were stored in the deep freezer (–80 °C) to slow oxidation and were used as a reference (Week 0) for both SPME-GC-MS and sensory analysis. Accelerated shelf life testing can be expressed using the *Q*_10_ concept. For the purpose of this study, an approximated *Q*_10_ value of 2 is used, meaning that the rate of oxidation doubled when the storage temperature increased by 10 °C.

### 2.4. Analysis of compounds

#### 2.4.1. Sample preparation

Unsalted and cheese & onion seasoned potato crisps were ground into small particles using a mortar and pestle (Fisherbrand™ Porcelain, UK). 2 g of the crushed sample was mixed with 5 ml ultrapure water in 20 ml amber headspace vials (Supelco, Bellefonte, PA, USA) and 100 μl of 0.01% 3-heptanone in methanol (internal standard) was added. Ultrapure water helps to promote the release of volatile compounds, especially in solid samples. The vials were hermetically capped with PTFE-faced silicone septa (Supelco, Bellefonte, PA, USA).

#### 2.4.2. Headspace SPME-GC-MS

The volatile flavour compounds associated with the cheese & onion seasoned potato crisps and the development of lipid oxidation markers in the unsalted potato crisps during accelerated storage were evaluated by the headspace gas analysis performed using solid phase micro-extraction (SPME) and GC-MS. The analysis was performed with an ISQ Single Quadrupole Mass Spectrometer, paired with a TRACE 1300 GC system, equipped with a ZB-WAX column (30 m × 0.25 mm I.D. × 1μm film thickness) and a TriPlus RSH autosampler (Thermo-Fisher Scientific, Waltham, MA, USA). A fused silica fibre coated with a 50/30 μm layer of divinylbenzene–carboxen–polydimethylsiloxane (DVB/CARBOXEN/PDMS; Supelco) was used to analyse headspace samples. The fibre was exposed to the headspace for a total extraction time of 20 min at 70 °C. After extraction, the fibre was immediately thermally desorbed at 250 °C for 4 min. The oven temperature was as follows: 40 °C for 2 min, then to 240 °C at 6 °C min^–1^, held for 5 min. MS was operated in the electron impact (EI) ionisation mode at 70 eV and data acquisition was achieved at a scan rate of 0.20 s^–1^ over an *m*/*z* range of 35–300. The peak area was processed with Xcalibur Software and identification of aroma compounds using NIST library software (NIST/EPA/NIH Mass Spectral Library, version 2.0, Faircom Corporation, U.S.).

### 2.5. Sensory method

Sensory analysis was aimed to monitor the change in sensory perception of flavoured potato crisps with increasing storage time. For this purpose, the seven-point hedonic scale was used as reported in Meilgaard *et al.*, 1999.^26^ Ten assessors (six female and four male) was selected from Pipers Crisps Ltd (UK) and all the assessors were trained following the ISO standard guidelines, ISO 8586:2012.^27^ All assessors were experienced in discrimination and descriptive tests and had worked previously with all the sensory attributes for flavoured potato crisps. Full approval of a local ethics committee was obtained before the study commenced. Informed consent was obtained from all assessors after the nature of the methods and nutritional consumption per session was fully explained.

Deep fried flavoured potato crisps were stored at 45 °C in an oven and samples were removed after 1, 2, 3, 5 and 7 weeks to monitor the impact of ageing on the sensory perception of crisps, and samples were stored at –80 °C until being evaluated by trained panellists. The testing was carried out in two different sessions. At each session, the assessor received a different potato crisp and reference sample (fresh crisps produced on the same day as the test samples and stored at –80 °C was considered as a reference sample). For each sample, the assessor compared the 3-coded shelf-life sample with reference and rated the magnitude of difference of the coded samples from the reference sample using a seven-point category scale anchored as follows: 1 = very big difference, 2 = big difference, 3 = moderately big difference, 4 = moderate difference, 5 = slight difference, 6 = very slight difference and 7 = no difference.^26^ A range of sensory attributes was used to monitor the impact of ageing with or without N_2_ gas flushing and seasoning such as crisps appearance, crisps colour, crisps oiliness, flavour coating, flavour colour, flavour odour, crisps texture, potato taste, rancidity, and seasoning perception such as salt, cheese and onion. The order of presentation was randomised within a session and all panellists were required to cleanse their palate using provided water (Evian, France) between the test samples and 5 min break after 2 sets of samples. The sensory session was conducted at ambient temperature in a well-lit room.

### 2.6. Data analysis

All samples were analysed in triplicate. Data were analysed using both Design Expert (Stat-Ease, Inc., USA) and XLSTAT 2017 (Addinsoft, USA), using analysis of variance with Tukey's *post hoc* test (*P* < 0.05) to identify significant differences between sample sets. The sensory panel's scores were subjected to analysis of variance and the stored samples means were compared to that of the control by Dunnett's test for multiple comparisons with a control. The critical differences between the means of the stored samples and the control were computed for one-tailed Dunnett's test, especially as the stored crisp samples will have slightly higher difference than their fresh counterpart. Principal Component Analysis (PCA) was performed using Excel XLSTAT Version 2017 (results presented in the ESI†).

## 3. Results and discussion

### 3.1. Volatile compounds in potato crisps using Headspace SPME (HS-SPME)

The volatile profile of both unsalted and cheese & onion seasoned potato crisps determined by SPME-GC-MS is shown in Table 1; many volatile compounds were present in both products, where some that are associated with the base (unsalted) potato crisps are commonly recognised as secondary markers for lipid oxidation over storage (Table 1). These volatile compounds include aldehydes such as hexanal, heptanal, nonanal, octanal and 2-pentyl furan, which are produced by the heterolytic acid decomposition of hydroperoxides of methyl linoleate or oleate in high oleic oils.^6^,^7^ A number of studies have shown hexanal to be a primary indicator of secondary lipid oxidation, which is commonly found in potato crisps and as a typical undesirable off-flavour.^5^,^11^,^28^,^29^ Other volatile compounds such as aldehydes, disulphides and terpenes (essential oils naturally present in seasoning) were observed specific to the flavouring in the samples, and were observed in the cheese & onion seasoned potato crisps (similar volatiles were reported with dairy food products^30^–^32^).

**Table 1:** Sensory descriptors of volatiles compounds found in the unsalted potato crisps and cheese & onion (C & O) seasoned potato crisps

Compound	Unsalted	C & O	Sensory description
Hexanal	×	×	Green, leafy and grassy^33^,^35^,^42^
Heptanal	×	×	Oily, fatty, citrus^35^,^42^
Nonanal	×	×	Oily, fatty, citrus^41^
Octanal	×	×	Oily, fatty, soapy, citrus^36^
2,4-Decadienal	×	×	Fatty, chicken, aldehydic, green, fried and potato^34^
2-Decenal	×	×	Waxy, fatty, earthy^34^
Benzaldehyde	×	×	Pleasant almond-like odour^34^
Phenylacetaldehyde	×	×	Honey, sweet, floral, chocolate, cocoa, spicy^34^
2-Pentyl furan	×	×	Green, waxy, with musty^35^
2-Methyl butanal		×	Musty, furfural and rummy, nutty, cereal, caramel and fruity^37^
3-Methyl butanal		×	Fruity dry green^37^
*n*-Propyl acetate		×	Estry, fruity^45^
Dipropyl_disulfide		×	Sulfurous, alliaceous, fresh, green onion^35^,^46^
Diallyl_disulfide		×	Sulfurous, onion, green onion^35^,^46^
Methyl-1-propenyl disulfide		×	Garlic, onion^35^
Isopropyl propyl disulfide		×	Alliaceous, onion, meaty, sulfurous, cabbage^35^
Alpha-pinene		×	Sweet, citrus, terpenic^36^
Limonene		×	Orange, citrus^36^,^47^
Methyl pyrazine	×	×	Nutty, brown, roasted, musty and astringent^38^
2,5-Dimethyl-pyrazine	×	×	Baked and nutty^35^
Trimethyl pyrazine	×	×	Raw, musty, nutty, potato^38^,^39^
3-Ethyl-2,5-dimethyl-pyrazine	×	×	Roasted and baked^38^

#### 3.1.1. Lipid oxidation in unsalted and seasoned potato crisps

**Fig. 1:**
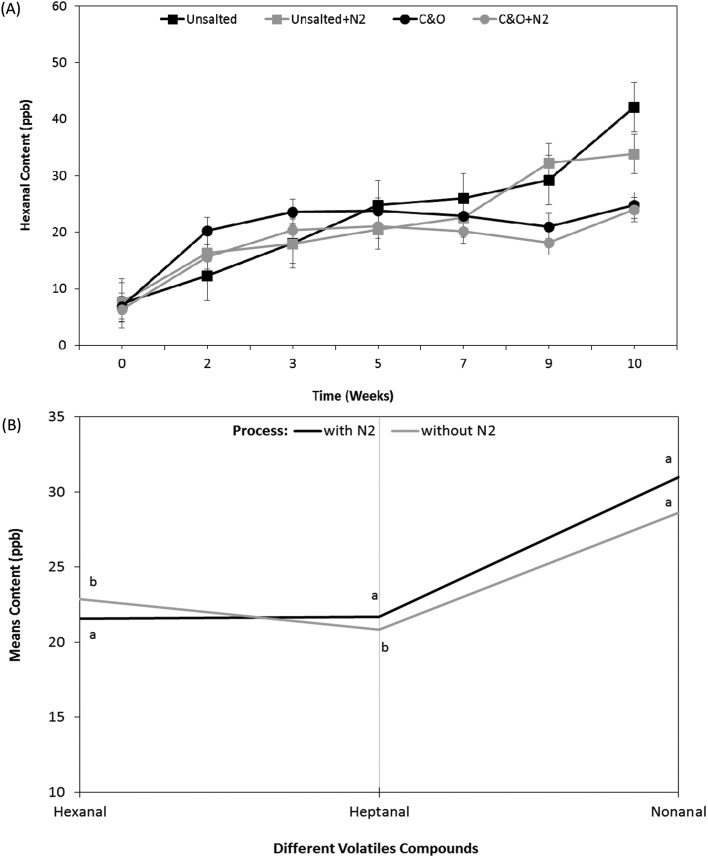
(A) Hexanal concentration (ppb headspace) in unsalted potato crisps and cheese & onion (C & O) seasoned potato crisps with and without N_2_ gas flushing during the accelerated storage at 45 °C for 10 weeks. (B) Impact of nitrogen gas flushing on headspace volatiles profiles of the unsalted potato crisps stored for 7 weeks at 45 °C.

In this study, hexanal was used as a primary indicator for the autoxidation of the oils.^13^,^14^ The hexanal content was in a similar range (between 6 ppb and 40 ppb with increasing storage time) in both cheese & onion and unsalted potato crisps (Fig. 1A). A statistically significant difference was observed between the unsalted crisps with and without nitrogen (N_2_) gas flushing in terms of hexanal content, where higher lipid oxidation was reported with unsalted crisps without N_2_ flushing as compared to the nitrogen-flushed samples (unsalted + N_2_) and is shown in Fig. 1A (*p*-value <0.0001).

The hexanal content in unsalted potato crisps fried in HOSO with N_2_ gas flushed was significantly lower than in unsalted potato crisps without N_2_ flushing (*p*-value <0.001); a similar increase in hexanal content was reported by Marasca *et al.*, (2016) with unsalted crisps fried in 100% normal sunflower oil with/without N_2_ gas flushing.^29^ Similar behaviour was observed with other volatile compounds associated with lipid oxidation such as heptanal and nonanal (shown in Fig. 1B). A significant increase in hexanal content was evident after 7 weeks of storage with N_2_ flushing, whereas unsalted crisps without N_2_ flushing showed a significant increase after 6 weeks of storage. Despite the effectiveness of the N_2_ gas flushing, the unsalted crisps showed an increase in stability by 1 week; however rancid taste perception was reported by the sensory panel (discussed in detail later). The following results highlight that even with modified atmosphere packaging *i.e.* N_2_ gas flushing there is a threshold after which the lipid oxidation is inevitable with compromised sensory perception. However, a combination of both HOSO frying oil and N_2_ gas flushing showed significant impact on improving the shelf life stability of deep-fried unsalted (base) potato crisps.

Interestingly, the content of volatiles associated with lipid oxidation, *e.g.*, hexanal, in cheese & onion seasoned potato crisps packed with N_2_ gas flushing was stable up to 9 weeks and showed a significant increase in week 10 (*p*-value <0.0001) on storing at 45 °C (Fig. 1A, ESI in Fig. S1A†). A significant difference (*p*-value <0.05) was observed between cheese & onion seasoned potato crisps packed with and without N_2_ gas flushing (Fig. 1A). It appears that the presence of different ingredients in the cheese & onion seasoning slows the lipid oxidation process, hence reducing the hexanal formation as compared to the unsalted potato crisps over storage time (Fig. 1A). This can be explained by the antioxidant properties of the milk protein in the seasoning increasing the shelf life of the potato crisps and the flavour. It is well reported that the addition of milk protein (caseins) or whey protein-based coating helps in reducing the browning of many food products such as apple and potato slices,^43^ also in preventing lipid oxidation in oil-in-water emulsions.^44^ Le Tien (2001) showed the effect of milk protein-based coatings on the browning reaction of sliced apples and potatoes and indicated that whey protein and milk protein in combination with CMC (carboxymethyl cellulose) is a better antioxidant than calcium caseinate.^43^ In this study, no antioxidant agent was added to the seasonings (evident from the supplier's product specification); hence it is safe to assume that the presence of milk protein in the seasoning resulted in slowing the lipid oxidation process. However, further research is needed to determine the mechanisms of these antioxidant effects of milk proteins on deep-fried products such as potato crisps.

#### 3.1.2. Change in volatile compounds in cheese & onion seasoned potato crisps

**Fig. 2:**
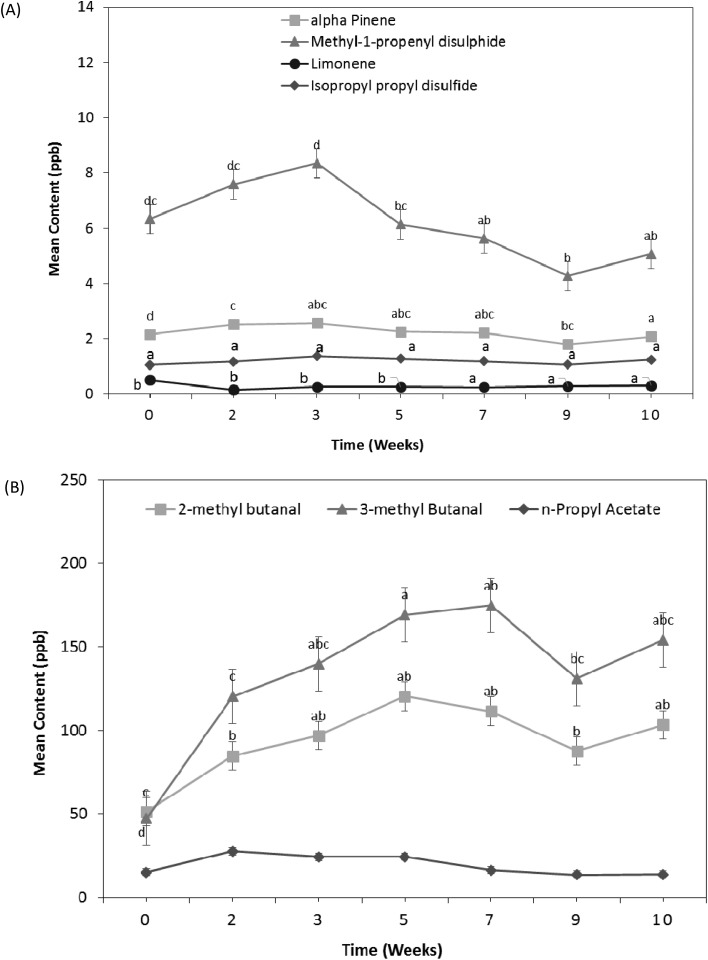
(A) Disulphide & terpene concentration (<100 ppb concentration headspace) and (B) aldehydes & acetates (>100 ppb concentration) in cheese & onion (C & O) seasoned potato crisps without N_2_ gas flushing during the accelerated storage at 45 °C for 10 weeks.

Besides the volatile compounds associated with autoxidation of oils and potato, a number of other volatile compounds were observed in the cheese & onion seasoned potato crisps. Volatile compounds such as limonene and alpha-pinene, sulphides such as dipropyl sulphide, dipropyl disulphide, dimethyl trisulphides, methyl-1-propenyl disulphide and isopropyl propyl disulphide and aldehydes such as 2-methyl butanal and 3-methyl butanal were identified with cheese & onion seasoned potato crisps (Table 1). These are proposed to be associated with the seasoning as similar volatile compounds were reported with other dairy or cheese products.^30^,^45^ These volatile compounds behaved differently with increasing storage time of the cheese & onion seasoned potato crisps (Fig. 2A and B).

##### Sulphides

**Fig. 3:**
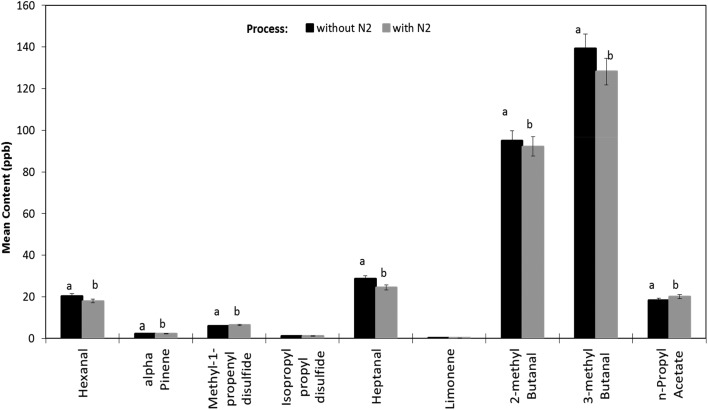
Impact of nitrogen gas flushing on headspace volatiles profiles of the cheese & onion (C & O) seasoned potato crisps after 10 weeks of storage at 45 °C.

A number of sulphides such as dipropyl sulphide, dipropyl disulphide, methyl-1-propenyl disulphide and isopropyl propyl disulphide were identified in cheese & onion seasoned potato crisps; these are generally associated with onion/garlic sensory perception.^35^ These volatile sulphide compounds associated with onion flavour were identified and reported in various studies.^35^,^40^,^41^ Dipropyl sulphide (garlic, onion), dipropyl disulphide (green onion), methyl-1-propenyl disulphide and isopropyl propyl disulphide concentrations decrease significantly (*p*-value <0.05) after 3 weeks of storage, evident in both with/without N_2_ gas flushing packaging (Fig. 2A and 3, ESI in Fig. S1B†). This decrease in disulphide compounds can be explained by the free-radical reactions in the presence of oxygen during lipid autoxidation (formation of lipid hydroperoxides), which may also catalyse the degradation of these sulfur-containing compounds and contribute to the decrease over storage time. Besides that, the decrease in disulphide content (with an increase in temperature) may be caused by the interchange of disulfide groups with sulfhydryl and disulfide groups of proteins or low proportion present in casein (in this case, cheese); a similar behaviour was reported with albumin.^46^

##### Terpenes

In the cheese & onion seasoned potato crisps 2 terpenes were identified *i.e.* alpha-pinene and limonene. Alpha-pinene (sweet, citrus, terpenic) showed a statistically significant decrease (*p*-value <0.05) after 9 weeks during shelf life storage at 45 °C (Fig. 2A). A slight increase (not statistically significant *p*-value >0.05) in limonene (citrus) content was evident with an increase in storage time (Fig. 2A). A significant difference (*p*-value <0.05) in alpha-pinene content was observed between cheese & onion seasoned potato crisps packed with/without N_2_ gas flushing, whereas no statistically significant difference (*p*-value>0.05) was observed with limonene (Fig. 3). This decrease in terpene content can be explained by the minor oxidation process in the presence of atmospheric oxygen and elevated temperature. A similar effect of atmospheric oxygen and temperature was reported with terpenes present in lemon oil.^47^ Nguyen (2009) reported a decrease in both terpenes associated with compressed lemon oil stored with and without N_2_ gas flushing: upon storage at 50 °C, a decrease in limonene and alpha-pinene with an increase oxidised products was observed.^47^

##### Aldehydes and acetates

Finally, a couple of aldehydes and acetates associated with cheese & onion seasoned potato crisps were observed in the headspace GC-MS profile. Aldehydes such as 2-methyl butanal and 3-methyl butanal showed a significant increase with an increase in storage time at 45 °C (Fig. 2B). A statistically significant difference in aldehyde content was observed in seasoned crisps packed with/without N_2_ gas flushing. A higher content of both aldehydes was observed with crisps without N_2_ gas flushing as compared to crisps packed with N_2_ gas flush (Fig. 3 and ESI in Fig. S1B†). Besides aldehydes, *n*-propyl acetate (estery, fruity) was also observed, which showed a slight decrease with increase in storage time (not statistically significant *p*-value >0.05) (Fig. 2B).

The use of headspace volatiles profiles enables an intuitive characterisation and precise ageing process of the complex seasonings and well-seasoned products; this allows a statistical comparison of cheese & onion seasoning aromatic volatiles profiles and impact of packaging environment on the volatiles/aroma stability. In accordance with the headspace volatiles profiles, the cheese & onions seasoning is a complex blend of different volatiles compounds responsible for specific aroma/sensory profile. Volatiles associated with seasonings such as aldehydes, acetates, sulphides and terpenes showed a noticeable decrease with increasing storage time and a significant impact was observed with gas-flushing on the volatiles stability. General pathways for the formation of different volatiles compounds and off-flavour in the different cheese varieties are well reported.^32^ Besides the aging/degradation of seasoning with increasing shelf life, the fact that the cheese & onion seasoning helps in slowing the lipid oxidation in deep-fried potato crisps pointed the possibilities of controlling both shelf life stability of the seasoning as well as the seasoned product, potentially incorporating milk proteins (or maybe other plant-based proteins) in seasonings. However, this may present some challenges such as the impact of protein on flavour release in other seasonings such as salt & vinegar seasoning (which is another popular seasoning in the UK), spicy seasonings such as sweet chilli, *etc.*; this may need further investigation and is subject to future studies. The shelf life stability profiles of these volatiles compounds correlate well with sensory perception of the well-seasoned potato crisps and it is also evident that the shelf life of the unsalted potato crisps may differ from that of well-seasoned potato crisps.

### 3.2. Sensory analysis

**Fig. 4:**
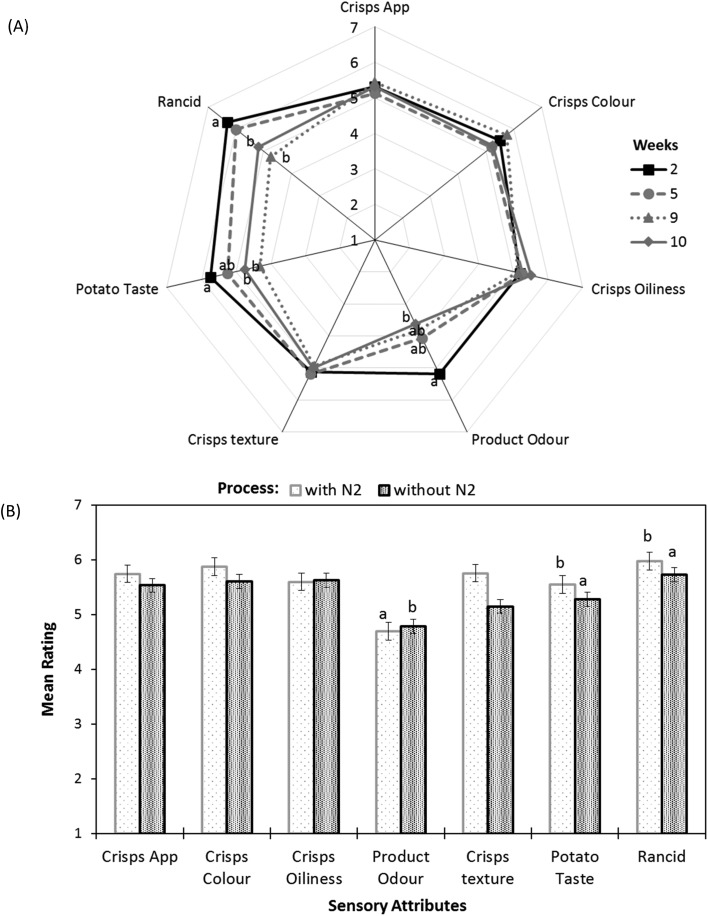
Sensory analysis of unsalted potato crisps showing (A) effect of accelerated storage time packed without N_2_ gas flushing, and (B) impact of nitrogen gas flushing after 7 weeks of storage at 45 °C.

For the unsalted potato crisps, the trained sensory panel perceived that the product odour, potato taste and rancidity were significantly affected by the storage time at 45 °C (Fig. 4A). Product odour (in terms of stale and off-taste), potato off notes and rancidity were significantly increased (*p*-value <0.05) after 7 weeks of storage at 45 °C when unsalted crisps were packed with N_2_ gas flushing. A significant difference was observed in terms of product odour, potato taste and rancidity sensory perception between unsalted potato crisps packed with or without N_2_ gas flushing (Fig. 4B). Unsalted potato crisps packed with N_2_ gas flushing perceived less rancidity and potato taste (stale/off-notes) as compared to potato crisps packed without N_2_ gas flushing (*p*-value <0.05) after 7 weeks of storage; however, a panellist can still perceive rancidity while comparing with reference for crisps packed with N_2_ gas flushing. The following sensory behaviour correlates well with volatiles compounds observed in headspace GC-MS analysis with increasing storage time and packing atmosphere (with/without N_2_ gas flushing). A significant increase in rancidity and product odour (stale/off-notes) can be explained by an increase in volatile compounds associated with lipid oxidation such as hexanal, nonanal, octanal, *etc.*, whereas potato off-taste can be explained by volatiles associated with potatoes such as 2,4-decadienal, methyl pyrazine, 2,5-dimethyl pyrazine and trimethyl pyrazine. Pyrazines are generally associated with positive sensory perception in deep-fried potato crisps such as nutty, brown, roasted and baked, but also associated with negative sensory perception in potato crisps such as raw, musty and potato sensory perception (Table 1).

Headspace volatiles profiles showed a noticeable decrease (not significant *p*-value >0.05) in pyrazines such as 2,5-dimethyl pyrazine, methyl pyrazine and 3-ethyl-2,5-dimethyl pyrazine associated with nutty, roasted, baked sensory profiles with increasing storage time, whereas slight increase in trimethyl pyrazine (not significant *p*-value >0.05, data not shown) might be responsible for potato/raw off-notes.

**Fig. 5:**
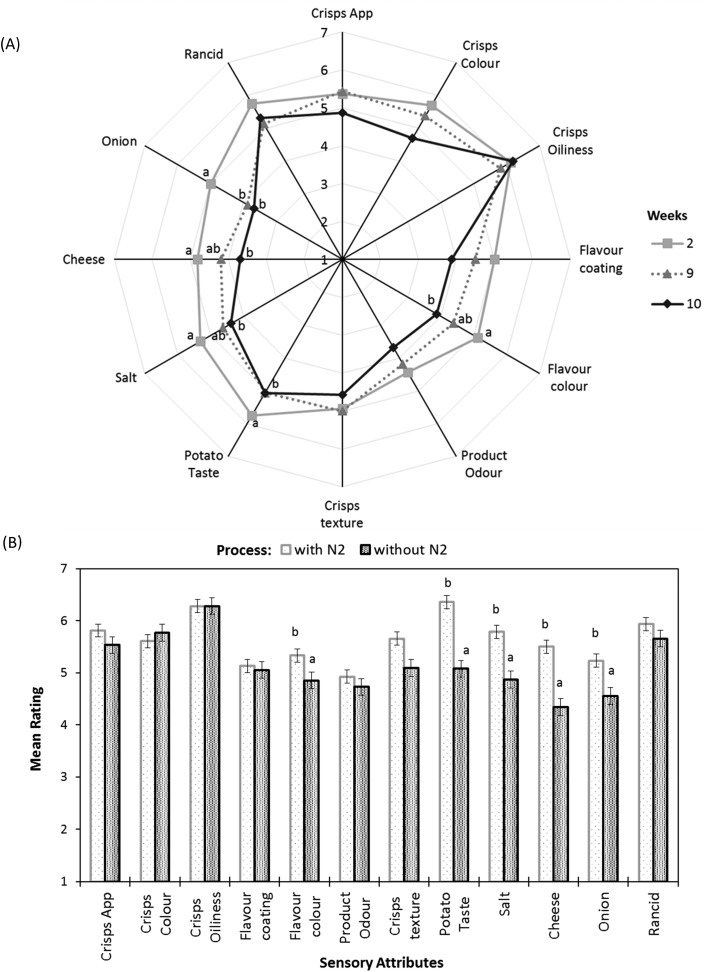
Sensory analysis of cheese & onion seasoned potato crisps showing (A) effect of accelerated storage time packed without N_2_ gas flushing, and (B) impact of nitrogen gas flushing after 7 weeks of storage at 45 °C.

Similar to unsalted potato crisps, for the cheese & onion (C&O) seasoned potato crisps, a significant difference was observed between crisps packed with and without N_2_ gas flushing (*p*-value <0.05, Fig. 5B). For cheese & onion seasoned crisps packed with N_2_ gas flushing, flavour/taste perception significantly decreased after 9 weeks of storage at 45 °C (*p*-value <0.05, Fig. 5A), whereas a decrease in flavour perception was evident after 6 weeks of storage when seasoned crisps were packed without N_2_ gas flushing. This decrease in flavour perception correlated well with the significant decrease in volatile compounds such as disulphide (associated with onion flavour) and terpenes (associated with cheese flavour with sweet, citrus and terpenic characteristics) in headspace SPME analysis. Comments such as stale/sweet stale and rancid were reported after 6 weeks of storage packed without N_2_ gas flushing by the trained sensory panel; this was also reflected in the rating of potato taste, flavour colour and crisps & flavour product odour (Fig. 5A, statistically significant *p*-value <0.05). These comments are likely associated with an increase in volatile compounds (such as hexanal, heptanal and nonanal) from autoxidation of oils and potato oxidation. Similar off-flavours were reported in various studies.^30^,^42^,^48^ Besides stale/off-taste, different flavour perceptions were also rated significantly low (*p*-value <0.001) in comparison with a reference (fresh samples) after 6 weeks of storage when samples were packed without N_2_ gas flushing and comments such as flavour fade was reported. These sensory ratings and comments can be explained by a significant decrease in volatiles compounds associated with cheese and onion flavour such as terpenes and disulphides (Fig. 2A, B and 3).

In summary, the shelf life stability of potato crisps and well-seasoned potato crisps depends on both autoxidation of oils, potato oxidation and flavour stability with storage time and package environment (oxygen content). For instance, considering the hexanal content as a function of time, the shelf life stability of unsalted potato crisps without N_2_ gas flushing is up to 6 weeks (24 weeks in real time) as compared to N_2_ gas flushing, which can extend the shelf life stability to 8 weeks (32 weeks in real time). However, the sensory perception in terms of stale, rancid and taste perception itself of the following products cannot be neglected. For example, product odour, off-taste of old potato and rancidity were beginning to be affected by storage time after 6 weeks of storage with both packaging environment (Fig. 5A and B).

Cheese & onion seasoned potato crisps without N_2_ gas flushing were stable to lipid oxidation for up to 7 weeks (38 weeks) whereas cheese & onion seasoned potato crisps packed with N_2_ gas flushing showed stable behaviour up to 10 weeks (40 weeks) at 45 °C storage temperature. However, a significant decrease in sensory perception was evident after 9 weeks of storage, which cannot be discounted. For example, in cheese & onion seasoned potato crisps, even though the lipid oxidation markers such as hexanal or heptanal do not significantly increase with storage time at 45 °C (Fig. 1A), lower rating and a significant decrease in the product flavour perception led to lower rating (lower acceptability) by a sensory panel.

## 4. Conclusion

In summary, the shelf life stability of potato crisps and well-seasoned potato crisps depends on both autoxidation of oils, potato oxidation and flavour stability with storage time and package environment (oxygen content). In this study, a wide range of volatile compounds was identified in unsalted potato crisps and cheese & onion seasoned potato crisps. Cheese & onion seasoning slowed the rate of lipid oxidation and development of perceived stale/rancid flavour perception; this behaviour can be explained by the antioxidant properties of milk proteins present in cheese. Potato crisps fried in HOSO and packed in a controlled environment *i.e.* nitrogen gas flushing further improves the shelf life stability of the unsalted & C & O seasoned potato crisps. This research shows that differences can be seen in the oxidative stability of lipids in unsalted and cheese & onion seasoned potato crisps; however, further research is needed to determine the mechanisms of these differences.

## Conflicts of interest

There are no conflicts of interest to declare.

## Supplementary Material

Supplementary information
