# Supplementary material for: The impact of nitrogen gas flushing on the stability of seasonings: volatile compounds and sensory perception of cheese & onion seasoned potato crisps
Source: Food Funct. 2018 Aug 10;9(9):4730–41. doi: 10.1039/c8fo00817e (PMC6148464; doi:10.1039/c8fo00817e)
Supplement: Supplementary file 1 [file FO-009-C8FO00817E-s001.pdf]

Electronic Supplementary Material (ESI) for Food & Function.
This journal is © The Royal Society of Chemistry 2018

Supporting information

Supplemental Figures

(A)

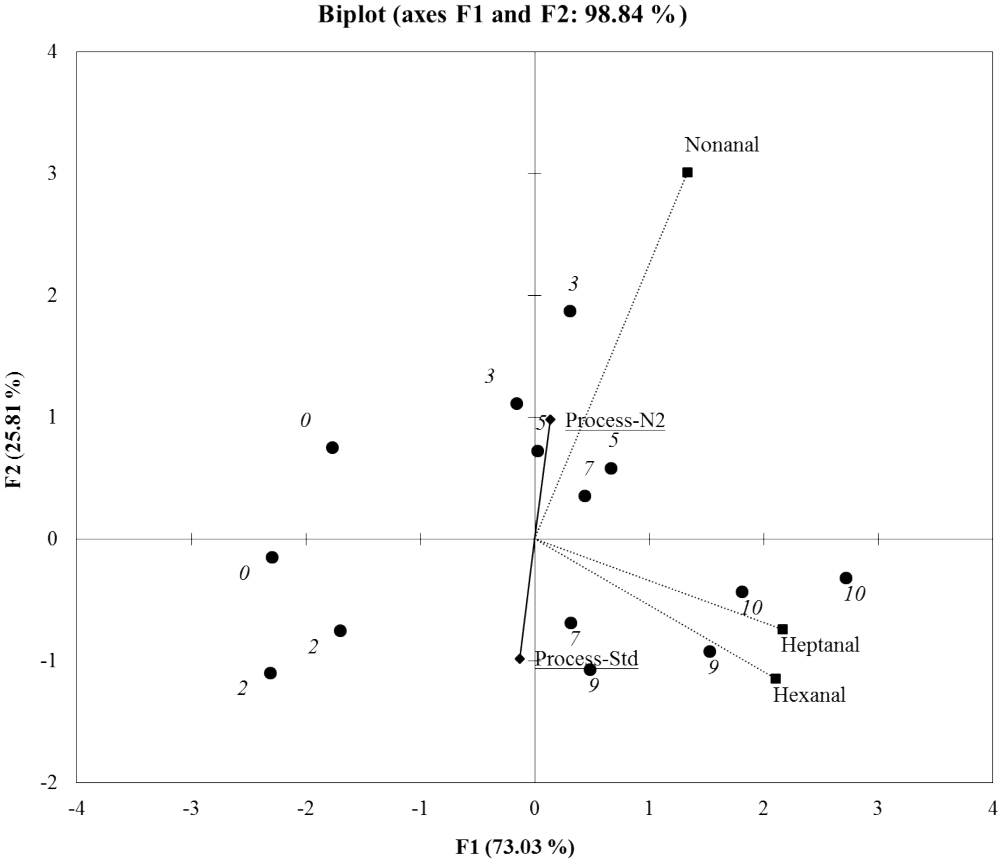

(B)

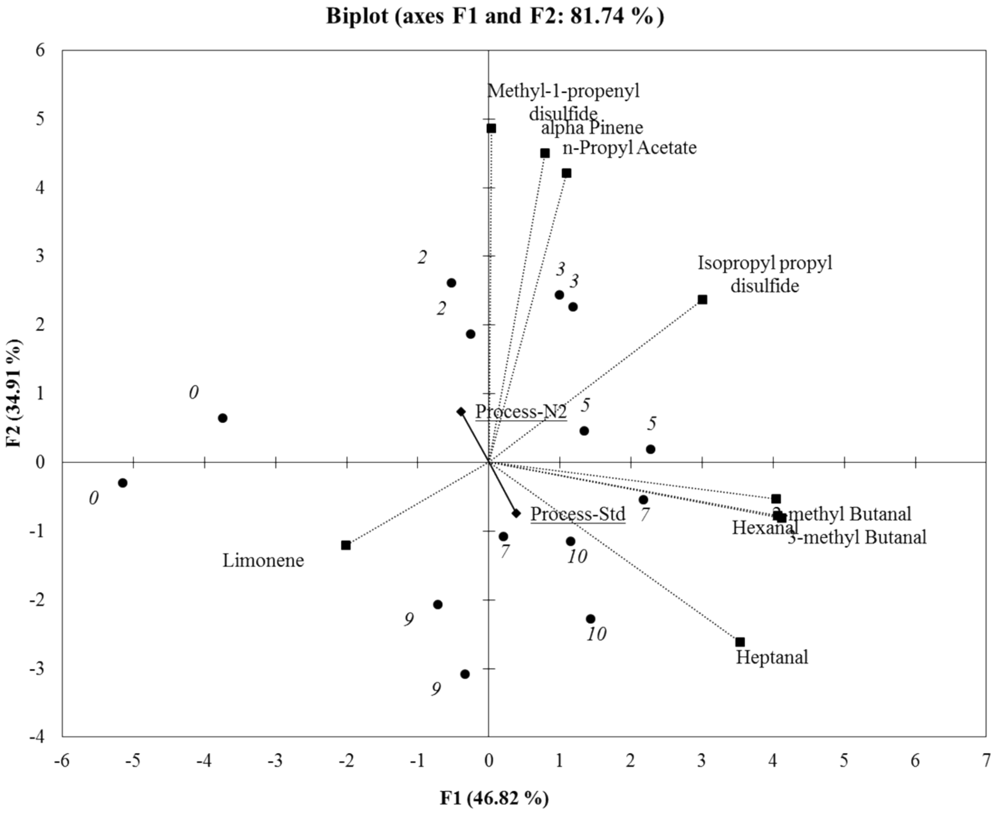

Figure S1: *Principal component analysis map (PC1 and PC2) involving the quantities of the significant components such as different packaging environment (underlined), storage time (italic) and volatile compounds attributes, where (A) Unsalted potato crisps, and (B) Cheese & onion seasoned potato crisps.*

Summary of all samples via Principal Component Analysis

Principal component analysis (PCA) was used to illustrate the difference between all volatiles associated with unsalted and C&) seasoned potato crisps with increasing storage time and controlled packaging environment. For unsalted crisps, the first principal component (PC1) accounted for 73.03% of the variance in the dataset and showed a trend with the amount of aldehydes from top to bottom and different storage time. The second principal component (PC2) accounted for 25.81% of the variance and showed separation between the N_2 gas flushing (top) and without N_2 gas flushing packaging (bottom). Similarly, for cheese & onion seasoned potato crisps, the first principal component (PC1) accounted for 46.82% of the variance in the dataset and showed a trend with terpenes, acetate and disulphides (top) and aldehydes (bottom) and different storage time. The second principal component (PC2) accounted for 34.91% of the variance and showed separation between the packaging environments *i.e.,* crisps packed with N_2 gas flushing (top) and crisps packed without N_2 gas flushing (bottom). Disulphides and terpenes were positively correlated and aldehydes such as hexanal, heptanal were negatively correlated. There were visible differences between packaging environment (*i.e.* with or with N_2 gas flushing) with both unsalted and C&O seasoned potato crisps, characterised by these samples being located in different corners of the PCA plot.
